# Neurodevelopmental Disorders and Environmental Toxicants: Epigenetics as an Underlying Mechanism

**DOI:** 10.1155/2017/7526592

**Published:** 2017-05-08

**Authors:** Nguyen Quoc Vuong Tran, Kunio Miyake

**Affiliations:** Department of Health Sciences, Graduate School of Interdisciplinary Research, University of Yamanashi, 1110, Shimokato, Chuo, Yamanashi 409-3898, Japan

## Abstract

The increasing prevalence of neurodevelopmental disorders, especially autism spectrum disorders (ASD) and attention deficit hyperactivity disorder (ADHD), calls for more research into the identification of etiologic and risk factors. The Developmental Origin of Health and Disease (DOHaD) hypothesizes that the environment during fetal and childhood development affects the risk for many chronic diseases in later stages of life, including neurodevelopmental disorders. Epigenetics, a term describing mechanisms that cause changes in the chromosome state without affecting DNA sequences, is suggested to be the underlying mechanism, according to the DOHaD hypothesis. Moreover, many neurodevelopmental disorders are also related to epigenetic abnormalities. Experimental and epidemiological studies suggest that exposure to prenatal environmental toxicants is associated with neurodevelopmental disorders. In addition, there is also evidence that environmental toxicants can result in epigenetic alterations, notably DNA methylation. In this review, we first focus on the relationship between neurodevelopmental disorders and environmental toxicants, in particular maternal smoking, plastic-derived chemicals (bisphenol A and phthalates), persistent organic pollutants, and heavy metals. We then review studies showing the epigenetic effects of those environmental factors in humans that may affect normal neurodevelopment.

## 1. Introduction

Neurodevelopmental disorders are a group of conditions characterized by impairments of social skills or intelligence with onset in the developmental period. According to the Diagnostic and Statistical Manual of Mental Disorders, Fifth Edition (DSM-V), they include intellectual disability (Intellectual Developmental Disorder), communication disorders, autism spectrum disorder (ASD), attention deficit hyperactivity disorder (ADHD), specific learning disorders, and motor disorders [1]. In 2011–2013, the estimated prevalence for ASD and other neurodevelopmental disabilities in children aged 3 to 17 years in the United States were 2.24% (1 in 45) and 3.57% (1 in 28), respectively [2]. Alarmingly, The Autism and Developmental Disabilities Monitoring (ADDM) Network that surveils children aged 8 years showed that the prevalence of ASD has increased from 0.66% to 1.46% over 10 years from 2002 to 2012 [3, 4]. Similarly, the prevalence of ADHD in children from 3 to 17 years also showed a 25.64% increase from 7.8% to 9.8% from 2003 to 2015 [5, 6]. The reasons for the increasing trend in ASD and ADHD are still controversial; explanations include changes in diagnostic criteria, reporting methods, or other factors such as environment, culture, and social-economic status that may affect the prevalence of neurodevelopmental disorders [7–9]. A previous study found indeed that the changes in diagnostic criteria alone account for only 33%, and a combination of changes in diagnostic criteria and reporting methods account for 60% of the increase in prevalence of ASD [7]. The search for etiologic and risk factors of neurodevelopmental disorders remains therefore an urgent issue and calls for further monitoring and research.

In the 1980s, epidemiologic studies by Barker et al. in England and Wales found that areas with high ischemic heart disease mortality rates also had high infant mortality rates at the time when the observed generation was in its early childhood. A birth cohort study from 1911 by the same group found that the lower the birth weight, the higher the cardiovascular disease mortality rate, hypertension, and impaired glucose tolerance rate [10–13]. From these findings, Barker et al. proposed an “adult-onset of fetal origin” hypothesis called “Barker's hypothesis,” stating that a low nutritional environment in the fetal stage increases the risk of chronic diseases in adulthood, which is the concept underlying the Developmental Origin of Health and Disease (DOHaD) [14]. The DOHaD concept is that the fetal-childhood environment affects the risk of chronic diseases in adulthood, based on knowledge obtained from epidemiological assessments of birth cohort studies. Research on developmental biology and human and animal physiology showed that the environment of fetal and early childhood have strong effects on development, health maintenance, and incidents of disease [15]. Based on the DOHaD theory, obesity, diabetes, and mental illness tend to develop in children born from women who are exposed to starvation during wartime [16–18]. Furthermore, folate and vitamin deficiency in the mother during pregnancy increases the risk of neurodevelopmental disorder [19–21]. In addition to maternal malnutrition, maternal stress due to various reasons is associated with child behavioral problems including ASD and ADHD [22–25].

Not limited to maternal stress or malnutrition, epidemiological studies have linked maternal exposure to environmental toxicants and neurodevelopmental disorders, particularly ASD and ADHD [26, 27]. Here, we focus on reviewing the relationship between maternal exposure to environmental toxicants and neurodevelopmental disorders and propose epigenetics as the linking mechanism.

## 2. Epigenetics and Neurodevelopmental Disorders

Epigenetics is defined as “a stably heritable phenotype resulting from changes in a chromosome without alterations in the DNA sequence” [28]. Thus, epigenetic mechanisms rely on DNA methylation, histone modification, histone variation, or noncoding RNAs, which change the chromatin structure and consequently control gene expression. Many conditions that fall under the term “neurodevelopmental disorders” are related to epigenetic abnormalities, namely the Prader-Willi syndrome, Angelman syndrome, ICF syndrome, and Rett syndrome.

Genomic imprinting is the concept underlying epigenetics, introduced 20 years ago. Specifically, in genomic imprinting, only one allele of a gene is expressed, depending on its parental origin. To date, more than 70 imprinted genes are identified in the human genome, together with a number of related diseases named “imprinting disorders” [29]. The Prader-Willi syndrome and Angelman syndrome are two illustrative examples for imprinting disorders originating in the 15q11-13 imprinted region. In this region, normally, the paternally derived chromosome expresses several genes such as small nuclear ribonucleoprotein polypeptide N (*SNRPN*), *SNRPN* upstream reading frame (*SNURF*), small nucleolar RNA, C/D box 116 cluster (*SNORD116*), and melanoma-associated antigen (MAGE) family L2 (*MAGEL2*), while ubiquitin protein ligase E3A (*UBE3A*) is expressed from the maternally derived chromosome specifically in neurons. The Prader-Willi syndrome, a neurodevelopmental disorder characterized by a short stature, muscle tension reduction, overeating and subsequent obesity, diabetes, and personality persistence, is caused by mutation or deletion of the paternally derived chromosome or by a maternal uniparental disomy (both chromosomes 15 were derived from the mother) [30]. The Angelman syndrome, characterized by intractable epilepsy and severe developmental delay, is caused by a genomic imprinting abnormality of the 15q11-q13 region, similar to the Prader-Willi syndrome, but its pattern is reversed. The causative gene is the *UBE3A* gene, expressed on the maternal chromosome but not the paternal chromosome in neurons [31]. In addition to the loss of gene expression in the 15q11-13 region, which results in the Prader-Willi syndrome or Angelman syndrome according to the parental origin of the chromosome, overexpression of genes in this locus results in a different disorder: the 15q11-13 duplication syndrome (Dup15q). The duplication is almost always of maternal origin and the disease is characterized by hypotonia, speech disorder, behavior disorders, abnormal EEG, and developmental delay with other associated symptoms such as autism and seizures [32]. Very recently, a large whole-genome bisulfite sequencing (WGBS) analysis on human brain and neuronal cell culture model of Dup15q revealed a global decrease in DNA methylation in both CpG regions and long intersperse element 1 (LINE-1) repetitive elements [33]. Furthermore, when compared to control samples, gene ontology analyses in the same study found that the differentially methylated regions are enriched for genes related to cell adhesion, brain, calcium channel, and membrane, many of which are known to have functions at neuronal synapses and are related to ASD [33]. More importantly, the authors also observed overlapped changes in DNA methylation and gene expression between neuronal cells exposed to polychlorinated biphenyl (PCB 95), an environmental toxicant, Dup15 model cells, and Dup15q model cells exposed to PCB 95, demonstrating an effect of environmental toxicant on neuronal genes via epigenetic mechanism, which will be discussed later in this review. Together with previous studies performed on postmortem human brain tissue, the results have suggested that epigenetic alterations, together with genetic dosage, contributed to the changes in gene expression in the pathology of the disease [34, 35]. The fact that loss or enhanced function of these imprinted genes leads to developmental disorders implies the importance of proper gene expression in neurodevelopment.

Another congenital disease caused by epigenetic abnormalities that impact development is ICF syndrome. The name ICF stems from the main symptoms of the disease: immunodeficiency, instability of the chromosome resulted from centromere instability, and facial anomalies. In this disease, the heterochromatin region near the centromere of chromosomes 1, 9, and 16, which is normally strongly methylated, is hypomethylated by DNA methyltransferase (DNMT3B) deficiency, resulting in chromosome instability [36]. DNMT3B, together with DNMT3A, are considered de novo DNA methyltransferase, acting to add methyl group to the cytosine residues in DNA. In vitro knockout or knockdown of *DMNT3B* was resulted in hypomethylation of satellite 2 repeats, leading to centromeric instabilities [37, 38]. Using induced pluripotent stem cells (iPSCs) and WGBS, a previous study revealed the alteration of gene expression and hypomethylation of promoters and enhancers in genes related to ICF syndrome phenotypes [39]. Another study revealed that changes in gene expression during neuronal differentiation were similar between *DNMT3B* knockdown human embryonic stem cells (hESCs) and ICF patient-derived iPSCs and that *DNMT3B* knockdown caused hypomethylation at pericentromeric regions and X chromosome but not at promoter regions of divergent genes as analyzed by WGBS [38]. Of late, there are studies that signified the role of DNMT3B in the maintenance of intragenic CpG methylation to ensure proper mRNA transcription and regulate alternating splicing [40, 41]. Moreover, a disease caused by a genetic mutation of *DNMT3A* was reported, characterized by overgrowth and developmental disorders [42]. These findings suggest that DNA methylation enzymes have important functions in the development of the immune system and the cranial nervous system.

Rett syndrome (RTT), a disease characterized by repeated hand movements, epileptic seizures, staggering gait, and autistic tendencies, is a representative case of a developmental disorder caused by epigenetic abnormalities. Most of the cases of typical RTT are caused by a mutation in methyl CpG-binding protein 2 (*MeCP2*) located on Xq28 [43]. Thus, RTT is a dominant inherited X-linked disease that affects 1 : 10,000 female live births, while affected males become embryonic lethal. In the brain, MeCP2 is highly expressed and increases over time during development. It is not only abundant in neurons but also expressed in astrocyte and glia cells, which is suggested to play an important role in the pathology of RTT. MeCP2 was first described as binding to methylated cytosine, recruiting other proteins such as nuclear receptor corepressor (NCOR)-SMRT (a silencing mediator of retinoic acid and thyroid hormone receptors) and corepressor complex Sin3A/HDACs to form a repressor complex and suppress gene expression. In addition to its repressing function, our knowledge of MeCP2 has increased overtime, and it is now known that MeCP2 can interact with coactivator cyclic AMP-responsive element-binding protein 1 (CREB1) to activate the expression of its target genes, alternate splicing sites via an interaction with YB1, a Y-box transcription factor, or regulate microRNA (miRNA) processing by interacting with DGCR8 to prevent the formation of the Drosha-DGCR8 complex [44]. Specifically, among various brain cells, it regulates the expression of a number of genes involved in synaptic functions or brain development—brain-derived neurotrophic factor (*BDNF*), distal-less homeobox 5 (*DLX5*), inhibitor of differentiation (*ID*), corticotropin-releasing hormone (*CRH*), insulin-like growth factor binding protein 3 (*IGFBP3*), cyclin-dependent kinase like 1 (*CDKL1*), protocadherin beta 1 (*PCDHB1*), and protocadherin 7 (*PCDH7*) [45]—and regulates glutamatergic synapse formation in early postnatal development [46]. In recent years, using RTT patient-derived iPSCs, several studies have revealed the defects of MeCP2 mutant neurons as seen in mouse models—smaller soma size, reduction of the number of synapse and spine, altered calcium signaling, and electrophysiological defects [47, 48]—or proposed a role of astrocytes in RTT [49, 50]. On the other hand, not only the loss of normal MeCP2 function but also a gain in MeCP2 function was found to cause neurodevelopmental disorders [51, 52]. MeCP2 transgene mouse models recapitulated the phenotype of the MeCP2 duplication syndrome, showing an increase in anxiety and deficits in coordination, learning, and memory [53].

Taken together, epigenetic mechanisms play an important role in brain development, especially the methylation of DNA. Any disturbances in the establishment, maintenance, or reading of DNA methylation are associated with neurodevelopmental disorders. As mentioned above, maternal stress, malnutrition, and exposure to environmental toxicants could alter the normal development and are related to neurodevelopmental disorders. Moreover, it has been long thought that epigenetics is one of the underlying mechanisms of the DOHaD hypothesis [54], and epigenetic patterns are more susceptible to environmental stresses than genome sequences [55]. There is evidence that expose to various environmental factors, such as mental stress and malnutrition during fetal and neonatal periods which can induce alterations in the epigenomics of the offspring [56–61]. Therefore, epigenetic mechanisms, particularly DNA methylation, may be the link between environmental toxicants and neurodevelopmental disorders.

## 3. Environmental Toxicant Exposure and Neurodevelopmental Disorders

### 3.1. Experimental Evidence of Environmental Toxicant Effects on Neurodevelopment

#### 3.1.1. Maternal Smoking

Maternal smoking during pregnancy has been related to many adverse effects in offspring including decreased birth weight, congenital anomalies, smaller neonate head circumferences, and sudden infant death syndrome [62]. The prevalence of woman smoking at any time during pregnancy differs between countries, ranging from 5% to 19%, and about one fifth of these quit smoking in later stages of the pregnancy [63, 64]. Tobacco smoke contains thousands of compounds that may have neurotoxic effects; among those, nicotine is the one most widely investigated. Prenatal exposure to nicotine has been proven to cause abnormal cognitive and emotional behavior and attention deficits [65–67]. Studies with the purpose to find out the mechanism of the relationship between smoking and behavioral problems exist, but there is no agreement. In brief, rat models treated with nicotine and mimicking a coexisting intermittent hypoxia state showed that nicotine-induced hypoxia reduces the expression of cyclin-dependent kinase 5 (*Cdk5*), an indispensable gene in the central nervous system, and plays a critical role in neurodevelopment [68] and delayed neuronal migration [69]. Another study has shown that prenatal nicotine exposure only impairs neurogenesis but not neuronal migration as observed by reducing the number of glutamatergic neurons in the medial prefrontal cortex and decreasing and disrupting cell cycles of neural progenitor cells in the ventricular and subventricular zones [70]. Recently, using tobacco smoke extract (TSE) to better demonstrate the effects of tobacco smoke, studies have suggested that TSE may have different effects on neurodifferentiation compared to nicotine alone [71]. Using PC12 cells in a neural differentiation model, a study showed that TSE exerted higher effect on inhibiting DNA synthesis and reducing cell proliferation compared to equivalent concentration of nicotine alone in undifferentiated cells, while in differentiated cells, TSE promoted the growth of dopaminergic phenotypes [72].

#### 3.1.2. Plastic-Derived Chemicals: Bisphenol A and Phthalates

Bisphenol A (BPA) is a chemical predominantly polymerized or modified and used in the plastic industry; thus, it is used in a wide range of consumer goods and commodities, especially in daily used food and beverage containers, dispensers, baby bottles, microwave cookware [73], or in healthcare-related products such as medical devices [74] or dental composite resins [75]. However, incomplete reaction or degradation of the polymer may release residual monomers of BPA. Human exposure to BPA is widespread, Centers for Disease Control and Prevention (CDC) reported BPA found in the urine of more than 90% of the US population, and more importantly, the concentration is higher in children (available in https://www.cdc.gov/exposurereport/). BPA has been proven to disrupt the endocrine system by interacting with estrogen, androgen, or thyroid hormone receptors [76, 77] and its effects on the reproductive system have been widely investigated [78]. In addition, perinatal exposure to BPA reduces synaptogenesis and synaptic proteins, alters the structure of synapse, affects behavior, and impairs learning-memory in male mice [79, 80]. Neonatal and perinatal BPA exposure affects postnatal gene expression and morphology of sexually dimorphic regions in the rat hypothalamus [81, 82]. In addition to effects on neurons, in the prefrontal cortex of the rat, adolescent exposure to BPA decreases the number of microglia in male rats and increases the number of microglia in female rat in adulthood, but does not affect the number of neurons or astrocytes, suggesting a long-term sex- and cell type-dependent effect of BPA [83].

Together with BPA, phthalates, chemicals used in the plastic industry, are also known to disrupt the endocrine system [84]. Based on their molecular weight and chemical properties, phthalates are classified into 2 subtypes that have different usage. High molecular weight phthalates including di-2-ethylhexyl phthalate (DEHP), butylbenzyl phthalate (BBzP), di(n-octyl) phthalate (DOP), diisononyl phthalate (DiNP), and diisodecyl phthalate (DiDP) are used in food containers, flooring, and wall covering and in medical tubing. Low molecular weight phthalates, dimethyl phthalate (DMP), diethyl phthalate (DEP), and dibutyl phthalate (DBP) are used in personal care products [84] or the coating of some medications [85]. Moreover, an earlier study has shown that BPA and phthalates may leak from food containers and enter the human body, as the usage of food that is not canned or packaged in plastic reduces urine levels of BPA and phthalates significantly [86]. While BPA and phthalate exposure is widespread, more importantly, phthalates have been proven to pass to the baby through the placenta and breast milk [87, 88]. An earlier study has claimed that DEHP added to the diet of mice has negative effects on behavioral tests of the offspring, although the author discussed that the results in some tests may not be the consequence of DEHP. Furthermore, the author debated that the concentration of DEHP in the environment may not be the cause for adverse effects in humans [89]. It is however known that DEHP is metabolized into mono-2-ethylhexyl phthalate (MEHP), and the effects of MEHP may therefore represent the toxic effects of DEHP. In a differentiation model of PC12 cells using nerve growth factor (NGF), MEHP exposure for 4 days enhanced neurite outgrowth induced by NGF [90]. MEHP upregulated choline acetyl transferase (ChAT) mRNA, a marker for cholinergic neurons, while it downregulated tyrosine hydroxylase (TH), a marker for dopaminergic neurons, suggesting that exposure to MEHP affects neuron differentiation [90]. This result suggests an effect of phthalates, particularly MEHP, on midbrain dopaminergic neurons, which are implicated in ADHD or schizophrenia (reviewed in [91]). In the developing rat brain, maternal exposure to DEHP decreases the concentration of essential lipids, particularly free cholesterol and sphingomyelin, as well as the mono- and poly-unsaturated fatty acid lipid composition, which plays an important role in neurodevelopment [92]. In respect to low molecular weight phthalates, prenatal and postnatal DBP exposure upregulates aromatase, an enzyme that has an important role in reproduction and neuroprotection, and downregulates estrogen receptor beta (ER*β*), which in turn reduces the expression of phosphate CREB and BDNF, two important neuroprotective proteins, in the rat hippocampus [93]. In line with these results, recently, in mouse neocortical neuronal cultures, treatment with DBP has been shown to impair the estrogen receptor pathway and induce neurotoxicity in a mechanism involved in the aryl hydrocarbon pathway [94].

#### 3.1.3. Persistent Organic Pollutants (POPs)

Persistent organic pollutants (POPs) have two main characteristics: resistance to environment degradation and accumulation in human or animal tissue. POPs are comprised of many different structures that have different toxicity [95]. Polychlorinated biphenyls (PCBs), a group of 209 related structures of chlorinate substituents on biphenyl rings, are used for heat resistance; organochloride pesticides (OCPs), such as hexachlorobenzene (HCB) or dichlorodiphenyltrichloroethane (DDT), are examples of POPs, many of them well-known as endocrine disruptors [96]. Exposure to POPs is widespread through environmental pollutants, daily used cosmetics, daily used items, or food. Importantly, POPs can be transferred from the mother to the child prenatally through the placenta [97] or postnatally by secreting into breast milk [98]. Therefore, prenatal and postnatal exposure to POPs may have many adverse health effects on the immune system and the reproductive system and may be related to other diseases such as cancer, diabetes, and obesity or to adverse pregnancy outcomes [98]. Moreover, since it is well-known that thyroid hormones are essential for neurodevelopment, prenatal and postnatal exposure to POPs could lead to neurobehavioral problems in children [99]. PCBs and OCPs have been suggested to be related to the pathogenesis of many neurodevelopmental and neurodegenerative disorders (reviewed in [100]). Experiments in rats exposed to PCBs during gestation and lactation showed increased activity of caspase 3 and DNA fragmentation, a marker for apoptosis, compared to control at postnatal day 1 but not at postnatal day 21 [101], suggesting that PCBs induce apoptosis in the developing brain. Another study in mice showed that low doses of non-dioxin-like polychlorinated biphenyls (NDL PCBs) given to the mother during the lactation period altered behavioral performances recorded during the mice's development [102]. Moreover, neurobehavioral toxicity of NDL PCBs increased from postnatal day 9 to postnatal day 28, but disappeared with increasing age, except for its effect on anxiety-related behavior, which was sex-dependent and permanent [102]. Newer chemicals that belong to POPs such as perfluoroalkyl acid (PFAA), perfluorooctanoic acid (PFOA), or perfluorooctane sulfonate (PFOS) were also shown to have neurotoxicity in mice or neuronal cell cultures by inducing apoptosis, inducing oxidative stress, and inhibiting neuronal differentiation [103–105], all of which may contribute to the changes in spontaneous behavior, habituation, and learning and memory observed in mice [106]. Exposure to PFOS and PFOA has also been shown to repress the expression of glutamine synthase-related genes and increase the extracellular level of glutamate in mouse primary astrocytes [105].

#### 3.1.4. Heavy Metals

Methyl mercury (MeHg) and lead are two representative heavy metals that have negative effects on neurodevelopment. Humans are widely exposed to MeHg through the consumption of contaminated seafood. The neurotoxicity of MeHg has been recognized since the incidence of Minamata bay, coining the term “Minamata disease.” The Minamata disease has occurred in humans who ingested fish and shellfish contaminated by MeHg discharged in waste water from a chemical plant. Children of women who had no symptoms of MeHg poisoning also showed abnormalities or neurodevelopmental problems [107]. Animal models have shown that gestational and lactational exposure to MeHg can cause significant deficits in behavioral tests and learning disabilities [108, 109], although a recent study showed that exposure to low doses of MeHg only affects the preadolescent but not the young adult period [110]. Experimental studies in animal or cell lines suggested that MeHg induced neurotoxicity by inducing oxidative stress, altering the kynurenine pathway and NMDA receptors, and impairing cytoskeleton instabilities [111–114]. Exposure to lead is also widespread as the sources may be air or water, and even a low level exposure to lead may cause negative effects in humans [115]. Prenatal and lactational lead exposure has been proven to affect learning and memory in mice by inducing pathological changes in the ultrastructure of synapses, downregulating synaptic genes, insulin-degrading enzyme (IDE) and insulin-like growth factor 2 (IGF2), and increasing beta amyloid 40 (Aβ40) and tumor necrosis factor- (TNF-) *α* [116–119]. Together with studies in mice, a study on human neurons revealed that exposure to lead increases the expression of serine/threonine protein phosphatases, which are associated with learning and memory [120].

Taken together, experimental studies indicate negative effects of environmental toxicants on neuronal cells and/or neurodevelopment; the mechanisms are however not fully understood, and certain findings are still controversial. Although some results are not directly related to any disease, the findings presented here support the hypothesis of DOHaD on neurodevelopmental disorders. For a complete evaluation of the DOHaD hypothesis in respect to neurodevelopmental disorders, cohort studies in human populations also have to be considered. Due to the large number of epidemiological studies on environmental toxicants, we only focus on several recent birth cohort studies that focused on those environmental toxicants described above.

### 3.2. Epidemiological Evidence for the Relationship between Environmental Toxicants and Neurodevelopmental Disorders

#### 3.2.1. Maternal Smoking

Several earlier reviews of studies from 1975 showed a relationship between tobacco smoke exposure and poorer academic achievement as well as an increased risk of mental retardation and neuropsychological and behavior problems; there were however still inconsistencies [121, 122]. Many studies observed a higher risk for ASD or ADHD symptoms in subjects prenatally exposed to environmental tobacco smoke (ETS) and have been reviewed elsewhere [63, 123–128]. Recently, several cohort studies in different countries supported the link between ADHD and prenatal tobacco exposure [129–131]. A mother-child cohort study of 1113 families in France that followed the child up to 5 years of age showed that maternal smoking predicted high symptoms of hyperactivity or inattention [129]. A Danish cohort study with a 7-year follow-up reported that not only maternal smoking but also the use of nicotine replacement products by the mother increased the risk for ADHD [131]. A Finish cohort study with more than 50,000 participants showed that, after adjusting for confounding factors, maternal smoking, no matter whether only in the first trimester or continuing after the first trimester, is associated with an increased risk for ADHD [130]. In another study that followed the child up to 15 years of age showed that maternal smoking of more than 10 cigarettes/day increased the risk for Tourette syndrome, Tourette syndrome comorbid with ADHD, and chronic tic disorders [132]. On the other hand, a case-cohort study of 633,989 children from parts of 11 US states and a recent meta-analysis of 6 cohort and 9 case-control studies found no relationship between ASD and ETS [133, 134].

#### 3.2.2. BPA and Phthalates

The relationship between plastic-derived chemicals and neurodevelopmental disorders is more complicated because of the sex-dependence of the latter. In a study of 244 mothers and their 3-year-old children, higher gestational BPA concentrations were associated with higher anxiety, hyperactivity, and depression scale scores, especially in girls [135]. However, in several later studies, prenatal BPA exposure was reported to have greater effects on boys [136] or have opposite effects on boys and girls [137–139]. Briefly summarized, higher BPA concentrations in the mother's urine samples during pregnancy were associated with higher scores on emotionally reactive, aggressive behavior and inattention symptoms that related to ADHD or conduct disorders. Prenatal phthalate exposure was also shown to be related to behavioral difficulties in children. In a Polish cohort study, prenatal phthalate exposure was inversely correlated with child psychomotor development such as cognitive, language, and motor abilities [140]. In line with this study, a Taiwanese cohort study showed that higher concentrations of DBP and DEHP in maternal urine samples were associated with externalizing disorders in children [141]. In contrast, in a Japanese cohort study, prenatal DEHP exposure did not lead to changes in infant thyroid hormone levels and had no adverse effects on neurodevelopment at early life stage [142].

#### 3.2.3. POPs

The relationship between POPs and neurodevelopmental disorders is also not clear. Several studies in Danish, Greek, and Faroese populations suggested no relationship between POPs and child neurobehavioral problems but a potential link with a reduction in cognitive abilities [143–145]. A German cohort study of polychlorinated dibenzo-p-dioxins and furans (PCDD/Fs) and PCB exposure suggested an effect of PCDD/Fs and PCB on attention performance in healthy children [146]. Another cohort study in a Japanese population showed that prenatal PFOA exposure had negative effects on the neurodevelopment of female infants at 6 months of age as assessed by Mental and Developmental Indices [147]. However, this study found no association between PFOA and neurodevelopment in infants at 18 months of age or PFOS at both 6 and 18 months of age, suggesting that the effects of POPs may be transient during early infancy.

#### 3.2.4. Heavy Metals

The negative effects of heavy metal on neurodevelopment are well described. A recent meta-analysis showed that increase of arsenic and manganese concentrations was associated with lower IQ, and prenatal exposure to manganese increased the risk of ADHD [148]. Other studies found that postnatal exposure to lead, which is measured by blood lead concentration, is associated with an increased risk for neurodevelopmental disorders and a decrease in cognitive scores [149, 150].

Overall, there is an abundance of cohort studies supporting the idea that exposure to environmental toxicants during pregnancy could result in neurodevelopmental disorders in children (summarized in Table 1). The molecular mechanism underlying the relationship between environment and neurodevelopmental disorders is however not clear. There is evidence suggesting that epigenetic mechanisms may constitute the link between environmental toxicants and disorders.

## 4. Epigenetic Alterations by Environmental Toxicants

### 4.1. Maternal Smoking

DNA methylation is the most well-known epigenetic mechanism involved in neurodevelopment [153]; thus, many studies on neurodevelopment focus on alterations of DNA methylation patterns (summarized in Table 2). DNA methylation is the process of adding a methyl group to the 5′ position of cytosine. This process mainly happens in a CG context, especially at regions rich in CG (CpG island), and catalyzed by DNA methyltransferase (DNMT) enzymes. DNMT3A and DNMT3B are known as de novo DNMTs that, in combination with DNMT3L, establish the methylation status. During cell division, DNMT1 and its associated proteins maintain the pattern of methylation (recently reviewed in [154]).

Brain-derived neurotrophic factor (*BDNF*) gene plays a key role in neurodevelopment, neurogenesis, and synaptic plasticity. BDNF has been linked to a number of neurologic disorders including neurodevelopmental disorders [155–157] and is now a gene of interest to investigate the neurological effects of environmental toxicants. Prenatal exposure to maternal cigarette smoking has been reported to induce a long-term effect on adolescent behavior possibly by inducing hypermethylation at *BDNF* promoter VI and 5′ untranslated region (UTR) [158].

Moreover, maternal smoking has been shown to alter the expression of genes related to neuropeptide signaling and signal transduction, as well as development, particularly tachykinin 3 (*TAC3*), left-right determination factor 2 (*LEFTY2*), heparin-binding EGF-like growth factor (*HBEGF*), mitochondrial fission factor (*MFF*), and fibulin1 (*FBLN1*), via mechanisms possibly related to genome-wide changes in DNA methylation [159]. A recent study showed that maternal smoking during pregnancy induced hypomethylation at CpG3 and CpG4 in total 13 CpG of the placental *NR3C1* gene, which has shown associations with newborn behavior [160]. Prenatal smoke exposure is known to result in a persistent effect on DNA methylation levels in adult peripheral blood granulocytes, as measured by repetitive element satellite 2 (Sat2) [161]. Epigenome Wide Association Studies (EWAS) provide a powerful tool for the identification of changes in epigenetics, specifically, in DNA methylation, induced by environmental toxicants [162]. One of the largest EWAS, screening 1062 newborn cord blood samples using Infinium Human Methylation 450 (HM450) platform, has identified hypomethylation of the aryl hydrocarbon receptor repressor (*AHRR*) and growth factor independent 1 transcription repressor (*GFI1*) and hypermethylation of cytochrome P450 1A1 (*CYP1A1*) and myosin IG (*MYO1G*) that were related to maternal smoking [163]. Later, another study focused on changes in the DNA methylation of the *AHRR* gene and found that the hypomethylation caused by maternal smoking was different between cord blood mononuclear cells, buccal epithelia, and placenta and that *AHRR* gene hypomethylation was maintained at 18 months [164]. Interestingly, the authors also reported that DNA methylation levels of monozygotic twins were more similar compared to dizygotic twins, suggesting that genetic variance has influences on the methylation level [164]. Another epigenetic mechanism that has been investigated in respect to neurodevelopment involves microRNA, an important regulator of gene expression that was found to have important roles in normal neuronal function and homeostasis [165]. Maccani et al. demonstrated that maternal cigarette smoking downregulated miR-16, miR-21, and miR-146a in the placenta, as confirmed in placental cell lines 3A, TCL-1, and HTR8 [166]. Later, the same group showed that high levels of placental miR-146a are associated with increased quality of movement scores; interestingly, high levels of placental miR-16 reduced attention scores in newborns [167]. In addition, smoking, together with other environmental toxicants such as BPA mixture, several POPs, and arsenic, were reported to alter transcription factor binding at promoter regions of miRNAs [168]. The study moreover pointed out that actin-dependent regulator of chromatin, subfamily a, member 3 (SMARCA3), and Forkhead Box P1 activated in embryonic stem cells (FOXP1_ES) were commonly enriched at promoter regions of miRNAs, suggesting that though different in responsive miRNA, environmental toxicants may have effect on similar transcription factor [168].

### 4.2. BPA and Phthalates

Most of the knowledge of DNA methylation alteration of BPA and phthalates comes from mouse models. A study on developing fetus mouse brains using spot DNA and methylation-sensitive quantitative PCR techniques showed that exposure to BPA was able to cause both hyper- and hypomethylation [169]. Intrauterine exposure to BPA was also reported to induce hypermethylation at *BDNF* promoter IV in a sex-specific manner [170]. Prenatal BPA exposure was reported to alter the level of DNMTs in mouse brain and this alteration may be sex-, region-, and dose-dependent. Briefly, gestational BPA exposure decreased DNMT1 but prevented female-specific reduction of DNMT3a in female mouse brains, while leaving the male mouse brain unaffected [171]. In line with this study, another study found that prenatal exposure to BPA decreased DNMT1 and DNMT3a in both the male and female mice prefrontal cortex and hypothalamus, but the alteration happened at different concentrations and at different trends between both sexes [172]. Furthermore, the authors also observed that changes in ER*α* and DNMTs were related to the changes in DNA methylation of the ER*α* (*Esr1*) gene [172], which was also observed in human breast cancer cells after DBP treatment [173]. A recent study reported for the first time that perinatal exposure to BPA induced hypomethylation at LINE-1 repetitive element in the human liver [174]. Given that LINE-1 is suggested to be a risk factor for schizophrenia or autism [175, 176], this finding suggests a link between BPA and other neurodevelopmental disorders via alteration of DNA methylation. Placental cell lines treated with BPA show an increase in miR-146a expression compared to untreated cell [177]. High concentration of phthalates in urine was found to be associated with a decrease in the expression of miR-185 in placenta [178]. In rat primary neuron cultures, exposure to BPA increased expression of *Mecp2* and MECP2 binding when reduced histone H3 lysine 9 acetylation at potassium chloride cotransporter 2 (*Kcc2*, *Slc12a5*) promoter, subsequently, repressed KCC2 expression [179]. It is well known that KCC2 is upregulated during neurodevelopmental period and has important roles in GABAergic function, neuronal plasticity, and formation of dendritic spine (reviewed in [180]).

### 4.3. POPs

Alterations of DNA methylation due to POPs in humans have been investigated in several studies, although those studies did not directly link to any disorders. The first study on changes in DNA methylation by POPs in a human population was performed using blood DNA of 70 Greenlandic Inuit and found a correlation between increased plasma concentrations of POPs and global DNA hypomethylation [181]. Kim et al. investigated changes in DNA methylation in Alu sequences in healthy Koreans and observed a similar correlation between a decrease in Alu DNA methylation and increased concentrations of POPs [182]. In accordance with these results, a later study by Itoh et al. showed that higher serum concentrations of POPs decreased global DNA methylation in a Japanese population [183]. In contrast, a study by Lind et al. on a population of 70-year-old Swedish found a relationship between high serum levels of POPs and global DNA hypermethylation [184]. Only one study examined PCB levels in human postmortem brains of patients with neurodevelopmental disorders and found that PCBs 95 was detected in 3/6 Prader-Willi syndrome postmortem brains and 5/6 postmortem brains with Dup15q. Moreover, this study reported hypomethylation of LINE-1 repetitive elements in postmortem brains of patients with Dup15q compared to control or ASD patients [185]. In consonance with this study, recent whole genome bisulfite study revealed an exceedance of hypomethylated genes toward hypermethylated genes in SH-SY5Y cells after long-term exposure to PCB 95 [33]. Moreover, of the 255 genes hypomethylated with long-term PCB 95 exposure, 209 and 201 genes are in common with SH15M, a cell model for Dup15q, and SH15M cell exposed to PCB 95, respectively. Furthermore, histone variant H2A.Z, which is also related to epigenetic mechanisms, was found to be enriched in the gene body and alleged to the transcriptional instability of those hypomethylated genes [33]. Noticeably, transcriptional instability induced by PCB 95 exposure was similar to the observation in Dup15q postmortem brain samples [34]. Altogether, these studies suggest epigenetic effects of POPs, particularly PCBs, in the pathology of neurodevelopmental disorders.

In rat primary hippocampal neurons, treatment with PCB 95 upregulated miR-132 and increased spine density [186]. Although the effect of PCB 95 seems to be positive, it is important to notice that increased spine density is also observed in patients with ASD [187]. Moreover, miR-132 is known to downregulate *MeCP2*, a key gene in neurodevelopment [188]; thus, the finding that PCB 95 upregulates miR-132 suggests a possible epigenetic mechanism behind PCBs neurotoxicity.

### 4.4. Heavy Metals

Exposure to heavy metals, particularly lead and mercury, is also known to alter DNA methylation. A study on methylation patterns in the promoter of p16, a gene related to neurodegeneration disorders, of 10 unexposed and 9 exposed individuals found that when unexposed group had an unmethylated pattern, the promoter of the p16 gene was partially methylated and fully methylated in the low-exposed and high-exposed groups, respectively [189]. A study reported for the first time that maternal exposure to lead was associated with alterations of DNA methylation in the umbilical cord blood [190]. In this study, the authors assessed maternal lead exposure using bone lead measurements and found a dose-response relationship between maternal patella lead concentrations and hypomethylation of LINE-1 repetitive elements and maternal tibia lead concentrations with hypomethylation of Alu sequence [190]. Regarding neurodevelopment, a study using mouse embryonic cortical neural stem cells found that MeHg treatment decreased mRNA levels of *Dmnt3b* but not *Dnmt1* and *Dnmt3a* and induced global DNA hypomethylation [191]. Interestingly, the changes in gene expression and DNA methylation patterns were also observed in daughter cells, even after the treatment with MeHg was stopped, suggesting an inherited possibility of epigenetic modifications [191]. A recent study using human embryonic stem cells, neural progenitor stem cells, and neurons showed that treatment with lead affected the neural differentiation process; global DNA methylation changes were observed including both hypermethylation and hypomethylation [192].

## 5. Epigenetic Inheritance

A critical question arising from the research presented here is whether epigenetic alterations induced by environmental toxicants propagate to later generations. Environmental factors affect not only the person who was exposed but also the next and further generations. This phenomenon in which the acquired phenotype is transmitted is called “transgenerational inheritance.” Classically, the constitutional change in the offspring is considered to be due to gene mutation. However, recent epigenetic studies suggest and support the idea that epigenetic changes could be inherited in the offspring, termed “transgenerational epigenetic inheritance” [193]. In fact, epigenetic modifications and mutations caused by environmental toxicants and nutritional disorders are maintained without being eliminated during the gametogenesis process and development after fertilization [194].

There are only a few, if any, studies that focus on the transgenerational epigenetic inheritance of environmental toxicant-induced alterations, especially, related to neurodevelopmental disorders. Although not spotlight environmental toxicants, animal models show that epigenetic effects caused by mental stress on parents can be transmitted to offspring. In addition, alterations of the epigenome in the neonatal brain induced by postnatal mental stress also occur in the spermatozoa. Furthermore, epigenomic alterations in brain and behavioral abnormalities were identified not only in children but also in grandchildren [195, 196]. A comprehensive review by Babenko et al. on animal experiments concluded that alterations of miRNA expression and DNA methylation in the placenta and the brain due to stress, which are linked to greater risks of schizophrenia, ADHD, autism, and anxiety- or depression-related disorders, are transmitted to later generations [196]. Moreover, the transgenerational epigenetic inheritance is not limited to mental stress or neurodevelopment disorders; exposure to environmental toxicants, which act as endocrine disruptors, also changes DNA methylation in spermatozoa and transmits these changes to the next generation [197–200]. A study by Manikkam et al. investigated the effect of plastics (BPA, DEHP, and DBP mixture), dioxin, pesticides, and hydrocarbons on reproductive diseases and showed that phenotypes and changes in the differential methylation regions are remained in the F3 generation [199]. A further study on plastic mixtures by the same group revealed that the incidence of obesity and reproductive disorders, but not kidney and prostate diseases, was increased in the F3 generation and that changes in the differential methylation regions associated with obesity-related genes (namely, *Tnfrsf12a*, *Esrra*, *Fgf19*, *Wnt10b*, and *Gdnf*) were observed in the sperm of the F3 generation [200]. This observation supports the idea of transgenerational epigenetic inheritance; it suggests however that only some diseases that have the underlying epimutation can be inherited. In another study, Marczylo and colleagues found that smoking induced changes in miRNA expression in the spermatozoa; importantly, many of those miRNAs have HDACs as the predicted target genes [201]. More recently, although not studied in humans, BPA exposure has been shown to downregulate ten-eleven translocation (*Tet*) in the testes of *Gobiocypris rarus* [202]. Given that the TET protein family plays a critical role in regulating DNA methylation and in development [203], the downregulation of TETs due to BPA exposure may relate to methylome maintenance, and further studies need to look at the changes in genes that have roles in establishing DNA methylation.

However, the epigenetic inheritance theory should be interpreted with some caution. One possible explanation for epigenetic inheritance is that environmental factors can affect not only the pregnant mother (F0) who has been exposed but also her fetus (child—F1) and even the primordial germ cells (grandchild—F2) in the fetus, so that it is not the inheritance but the changes in the grandchild that are under the direct effect of grandparent exposure. Thus, in order to demonstrate true epigenetic inheritance, it is necessary to determine whether the influence will remain until the next generation (F3 or more) of the grandchild [204]. In view of our recent knowledge about epigenetic mechanisms, there is not enough evidence for supporting a conclusion on epigenetic inheritance—which is different from the inheritance of changes in DNA sequences, and is therefore by some authors referred to as “soft inheritance” [205–207].

## 6. Conclusion and Perspective

In summary, a number of research have pointed out the relationship between in utero exposure to environmental toxicants and an increase in the risk of neurodevelopmental disorders; several lines of research describe the changes in epigenetic markers, mainly on DNA methylation. Although some studies reveal epigenetic changes in neurodevelopment-related genes [158, 172], it is unclear whether there is a relationship between the epigenetic alterations induced by environmental toxicants and the related neurodevelopmental disorders. The DOHaD hypothesis in particular is mainly based on cohort epidemiological studies and proposes epigenetics as its underlying mechanism. It is therefore important for further cohort studies to focus on epigenetic alterations of specific genes related to neurodevelopmental disorders, in order to clarify the etiological pathways. One study on oxytocin receptor (*OXTR*) gene methylation, for example, found that higher *OXTR* methylation at birth, which is associated with maternal abnormal behavior, psychopathology, and substance use, is related to higher callous-unemotional traits [208].

In addition to epidemiological findings, which will point out the suspected changes in epigenome, experimental studies are in need to endorse such changes which are the consequence of environmental toxicants and are the inceptions of neurodevelopmental disorders. These studies, however, will have to face difficulties in modeling the neurodevelopment especially in human, the underlying genome that may have influence on the susceptibility to environmental toxicants, as well as the demonstration for epigenetic changes at desired targets. Animal models have been used in studies for phenotypes, pathological changes, and the inheritability due to prenatal and perinatal exposure of environmental toxicants as reviewed in Section 3.1 of this paper. The existed mouse model for neurodevelopmental disorders (review in [209]) further supplied indispensable tools for investigating effects of toxicants on vulnerable genotype. Nevertheless, the differences in the underlying biology between mouse and human may deceive the epigenetic alterations after exposure to environment toxicants. The recent advances in iPSCs would provide a powerful material to overcome those barriers. Moreover, iPSCs have advantages in both directions: they can be reprogrammed from patients, and thus obtain the susceptible genotype; reversely, they can be differentiated into many cell types including neuronal cells and represent the process of neural development although the differentiation methods are still different between laboratories. A recent study, for instance, using iPSC found that chlorpyrifos, a potential POP [210], downregulated of neurogenesis genes during neural differentiation process [211]. Genome editing has been in research for decades and used productively; epigenome editing, on the other hand, is just in its beginning. Recent progresses have implicated the utility of CRISPR (clustered regularly interspaced short palindromic repeats) and Cas9 (CRISPR-associated protein 9) fused with epigenetic modifying enzyme such as TET1, DNMT1, or histone acetyltransferase p300 for epigenome editing [212, 213]. The application of these tools will provide strong evidences for the relationship between epimutations and diseases.

In this review, we discuss several environmental toxicants: tobacco smoke, plastic-derived BPA and phthalates, POPs, and heavy metals, which have been under investigation for decades. Hand in hand with industry developments, more and more toxicants are identified. Further studies are thus needed to investigate the relationship between newly identified environmental toxicants such as particulate matter (PM2.5) and polycyclic aromatic hydrocarbons (PAH), which were recently suspected to have a negative impact on fetal development [214]. In addition to prenatal environmental toxicant exposure, a recent review focused on neurodevelopmental disorders, particularly ASD, ADHD, and schizophrenia, and described that the susceptibility of neurodevelopmental disorders to toxicant exposure is not limited to the gestational period but extends into the postnatal period [215]. As described earlier, epigenetic mechanisms, especially DNA methylation, are assumed to be the mechanism underlying the effect of toxicants on neurodevelopment. In mammals, DNA sequences are methylated mostly at CpG sites and the reprogramming process of CpG methylation occurs during the prenatal period [216]. However, non-CpG methylation (CpA, CpC, and CpT) has also been detected, most abundant in stem cells and the brain of mice and humans, and found to have functions in regulating gene expression and possibly a role in genomic imprinting [217, 218]. Moreover, non-CpG methylation was found to be more abundant than CpG methylation in neurons and, interestingly, the percentage of non-CpG methylation sites increased during the postnatal period from 0 to 5 years [153]. Therefore, the role of non-CpG methylation in neurodevelopmental disorders is still unclear. As postnatal exposure to environmental toxicants also increases the risk of neurodevelopmental disorders, it is reasonable to consider the changes of DNA methylation at non-CpG sites under the effect of environmental toxicants. Lastly, as mentioned above, there is a lack of research focus on the transgenerational epigenetic inheritance of environmental toxicant-induced neurodevelopmental disorders. Gaining knowledge in the inheritability of epigenetic alteration by environmental toxicants will immensely aid in the progress of diagnosis and prevention of neurodevelopmental disorders.

## Figures and Tables

**Table 1:** Summary of recent reviews and cohort epidemiological studies on environmental toxicants and neurodevelopment.

Authors	Year	Toxicants	Type and subject	Assessments	Findings
Maternal smoking					
Kakbrenner et al. [133]	2012	Prenatal maternal smoking	Case-cohort study of 633,989 children from parts of 11 U.S states	ASD was based on surveillance program of the Autism and Developmental Disabilities Monitoring Network	Maternal smoking may not associate with ASD but may differ by ASD subgroup
Tran et al. [151]	2013	Maternal smoking during pregnancy	Finnish cohort study including 4019 ASD cases and 16,123 controls	Medical registry	Maternal smoking during the whole pregnancy was associated with an increased risk for pervasive developmental disorder (OR = 1.2, 95%CI: 1.0–1.5)
Zhu et al. [131]	2014	Paternal smoking and nicotine replacement use in pregnancy	84,803 Danish singletons, 50,870 children participated in the 7-year follow-up	Self-report; also observed for nicotine substitutes; ADHD was based on medical diagnosis and questionnaires	Children born to smoking or nicotine replacement usage mother and nonsmoking father have higher risk for ADHD compared to children born to smoking father and nonsmoking mother (adjusted hazard ratio HR = 1.63, 95%CI: 1. 36–1.94 and 2.28, 95%CI: 1.48–3.51 compared to 1.29, 95%CI: 1.14–1.47, resp.); children born to stop smoking mother and smoking father also increased risk of ADHD (HR = 1.70, 95%CI: 1.38–2.10; both maternal and paternal smoking during pregnancy associated with an elevated risk for ADHD, maternal smoking have more important effect
Melchior et al. [129]	2015	Maternal tobacco smoking in pregnancy	1113 families in France since pregnancy in 2003–2005 until the child's 5th birthday	Self-report; data collection at pregnancy, birth, 4, 8, 12, 24, and 36 months, and 5 years	Maternal smoking predicted high symptoms of hyperactivity or inattention OR = 1.95, 95%CI: 1.13–3.38 (in the 1st trimester); OR = 2.11, 95%CI: 1.36–3.27; (throughout pregnancy); maternal smoking throughout pregnancy have elevated level of hyperactivity or inattention OR = 2.20, 95%CI: 1.21–4.00; the dose of smoking showed a trend to associate with children's levels of hyperactivity or inattention OR = 1.49, 95%CI: 0.52–4.21 and 1.64, 95%CI: 0.53–4.64 for less than and equal to or higher than 10 cigarettes/ day, respectively
Tang et al. [134]	2015	Maternal smoking during pregnancy	Meta-analysis of 6 cohort and 9 case-control studies		No association between maternal smoking during pregnancy and ASD (pooled OR = 1.02, 95%CI: 0.93–1.13)
Joelsson et al. [130]	2016	Prenatal smoking exposure	Finnish cohort study of 10,409 ADHD cases and 40,141 controls	Self-report	Maternal smoking increased the odds for ADHD compared to entire samples when adjusted for confounder (OR = 1.73, 95%CI: 1.62–1.84); maternal smoking only in the first trimester or after the first trimester increased odds for ADHD, although smoking only in the first trimester had a lower risk (OR = 1.24, 95%CI: 1.03–1.50 in only the first trimester compared to OR = 1.79, 95%CI: 1.68–1.82 in group smoking after first trimester)
Browne et al. [132]	2016	Prenatal maternal smoking	Women early in pregnancy from 1996–2002, follow-up to when the child was 15 year old	Self-report and psychiatric diagnoses	Heavy maternal smoking (>10 cigarettes/day) increased risk for Tourette syndrome and chronic tic disorders; heavy maternal smoking also increased risk for Tourette syndrome comorbid with ADHD

BPA and phthalates					
Braun et al. [135]	2011	Gestational and childhood BPA exposure	244 mothers and their 3-year-old children	Spot urine samples; mother: twice during 16 and 26 weeks of gestation, and within 24 h after birth; child: 1, 2, and 3 years of age	Gestational BPA concentration were associated with higher anxiety, hyperactivity, and depression scale score; especially greater among girls at 3 years of age
Pepera et al. [137]	2012	Prenatal BPA exposure	198 African-American and Dominican mother and children pairs	Spot urine sample for mother during pregnancy and for child between 3 and 4 years old	In boys, high BPA concentration had higher scores on emotionally reactive and aggressive behavior (OR = 1.62, 95%CI: 1.12–2.32 and 1.29, 95%CI: 1.09–1.53, resp.); in girls, high BPA concentration had lower scores on emotionally reactive and aggressive behavior (OR = 0.74, 95%CI: 0.51–1.07 and 0.82, 95%CI: 0.70–0.97, resp.)
Evans et al. [136]	2014	Prenatal BPA exposure	153 mother-child pairs (children were 6 to 10 years old)	Maternal urine spot sample in the mean 26.6 weeks of gestation	Higher prenatal BPA concentration increased scores in some behaviors including ADHD or conduct disorders; the effect of BPA was worse in boys than in girls
Roen et al. [138]	2015	Prenatal BPA exposure	250 mothers and children	Spot urine sample for mother in the third trimester and for child between 3 and 5 years old	In boys, high BPA concentration were associated with higher behavioral symptom scores; when in girls, high BPA concentration were associated with lower behavioral symptom scores
Casas et al. [139]	2015	Prenatal BPA exposure	438 mother-child pairs (children followed up to 7 years old)	Spot urine samples in the 1st and 3rd trimesters	Prenatal PBA exposure was associated with increasing inattention symptoms in boys when decreasing inattention symptoms in girls
Polanska et al. [140]	2014	Prenatal and postnatal phthalate exposure	165 children in the Polish Mother and Child Cohort study	Phthalate levels in the urine	Prenatal phthalate exposure inversely correlated with child psychomotor development such as cognitive, language, and motor abilities
Ejaredar et al. [152]	2015	Prenatal exposure to phthalates	Systematic review of 11 articles		Prenatal exposure to phthalates is associated with adverse cognitive and behavioral outcomes in children from 0 to 12 years old, including lower IQ, and problems with attention, hyperactivity, and poorer social communication
Lien et al. [141]	2015	Prenatal phthalate exposure	122 mother-child pairs in Taiwan Maternal and Infant Cohort study	Mother: urine samples in the 3rd trimester; child: urine samples at 8-9 years of age	Higher concentration of DBP and DEHP in maternal urine samples were associated with externalizing disorders
Minatoya et al. [142]	2016	Prenatal phthalate exposure	224 participants (infants at 6 and 18 months of age)	Maternal blood MEHP concentration at 23–35 weeks of gestation	Prenatal DEHP exposure showed no changes in infant thyroid hormone level and had no adverse effects on infant neurodevelopment

POPs					
Strom et al. [143]	2014	Maternal exposure to PCBs and POPs	876 mother-child pairs in Danish cohort study	Maternal serum at 3rd trimester	No relationship between POPs and child neurodevelopment
Neugebauer et al. [146]	2015	Prenatal and postnatal exposure to polychlorinated dibenzo-p-dioxins and furans (PCDD/Fs), PCB, and lead	117 children	Maternal blood at 32 weeks of gestation and 1st week of breastfeeding	Prenatal exposure to PCDD/F and PCB significantly associated with attention performance in healthy children, whereas ADHD-related behavior remained unchanged
Kiriklaki et al. [144]	2016	Prenatal exposure to POPs	689 mother-child pairs in a Greece cohort study	Maternal serum in 2nd trimester of gestation	Prenatal exposure to POPs may be related to reduce cognitive but not to child behavioral difficulties
Oulhote et al. [145]	2016	Prenatal and postnatal exposure to POPs	656 children in Faroese cohort	Maternal serum at week 32 of gestation; child serum at age 5 and 7	Prenatal exposure to POPs had no association with behavioral difficulties in child; however, high serum PFAS concentration at ages 5 and 7 was related to behavioral problems
Goudarzi et al. [147]	2016	Prenatal exposure to PFOS and PFOA	428 mother-infant pairs	Maternal serum PFOS and PFOA concentrations	Prenatal PFOA exposure had negative effects on female infants at 6 months of age but not at 18 months of age. Prenatal PFOS exposure was not associated with neurodevelopmental scores

Heavy metals					
Rodríguez-Barranco et al. [148]	2013	Arsenic, cadmium, and manganese exposure	Meta-analysis		Increase of arsenic and manganese concentrations were associated with lower IQ, and exposure to manganese increases the risk of ADHD
Liu et al. [149]	2014	Postnatal lead exposure	1341 children	Lead: blood concentration at 3, 4, and 5 years of age	Higher blood lead concentration is associated with increase DSM-IV pervasive developmental problems
Rodrigues et al. [150]	2016	Postnatal lead exposure, prenatal and postnatal arsenic and manganese exposure	524 children in Bangladesh	Lead: blood concentration; arsenic and manganese: water concentration	There are relationships between higher blood lead concentration and water arsenic or manganese concentration with decrease cognitive scores

**Table 2:** Summary of studies of environmental toxicants with epigenetic alterations in human subjects.

Reference	Year	Environmental toxicants	Subjects	Findings
Smoking				
Maccani et al. [166]	2010	Maternal cigarette smoking	25 human placentas	Downregulation of *miR-16*, *miR-21*, and *miR-146a*
Toledo-Rodriguez et al. [158]	2010	Maternal cigarette smoking	156 adolescents	Hypermethylation at promoter 6 of *BDNF* gene and 5′ UTR
Flom et al. [161]	2011	Prenatal tobacco smoke	90 women	Demethylation at Sat2 repetitive elements
Suter et al. [159]	2011	Maternal smoking	36 placental samples	Changes in genome wide placental DNA methylation
Novakovic et al. [164]	2014	Maternal smoking	Cord blood mononuclear cells, buccal epithelia, and placentas	Induced hypomethylation at *aryl hydrocarbon* *receptor repressor (AHRR)* gene with differences between tissue and maintenance at 18 months of age
Stroud et al. [160]	2016	Maternal smoking during pregnancy	45 mother-infant pairs age 18–35	Demethylation at CpG3 and CpG4 of placental *NR3C1* promoter revealed that monozygotic twins are generally more similar in their DNA methylation level than dizygotic twins

BPA and phthalates				
Avissar-Whiting et al. [177]	2010	BPA treatment	Placental cell lines 3A, TCL-1, and HTR-8	Increase *miR-146a*
Kundakovic et al. [172]	2013	In utero BPA exposure	Cord blood samples	Sex-specific (males) hypermethylation at *BDNF* promoter IV
Faulk et al. [174]	2016	Perinatal BPA exposure	Human and mouse liver samples	Hypomethylation at LINE-1 repetitive element in human and mouse
LaRocca et al. [178]	2016	Prenatal phthalates exposure	Placentas	Decrease in *miR-185* expression

POPs				
Rusiecki et al. [181]	2008	Plasma POP concentration	Blood DNA of 70 Greenlandic Inuit	Increasing serum levels of POPs have a strong correlation with global DNA hypomethylation as assessed by Alu repetitive element
Kim et al. [182]	2010	Lipid-standardized concentrations of POPs	Blood DNA of 86 healthy Koreans	Decrease DNA methylation in the Alu sequence correlate with increase concentration of POPs
Mitchell et al. [185]	2012	Polychlorinated biphenyls (PCBs) and polybrominated diphenylethers (PBDEs) in human postmortem brain	Human postmortem brain	PCB 95 was detected in 5/6 Dup15q postmortem brain and may be related to hypomethylation of LINE-1 repetitive elements
Lind et al. [184]	2013	Lipid-standardized concentrations of POPs	524 70-year-old Swedish	Global DNA hypermethylation is associated with high levels of POPs
Itoh et al. [183]	2014	Serum organochlorine level	Leukocyte DNA of 399 Japanese	Inverse association between global DNA methylation and serum organochlorine level
Heavy metals				
Kovatsi et al. [189]	2009	Lead blood concentration	19 individuals (10 control and 9 exposed)	*p16* promoter was partially methylated under low exposure of lead and was fully methylated under high exposure of lead
Pilsner et al. [190]	2009	Prenatal lead exposure	103 umbilical cord blood	Maternal lead exposure was associated with hypomethylation of Alu and LINE-1 repetitive elements
Senut et al. [192]	2014	Lead	Human embryonic stem cells and hESC-derived neurons	Global DNA methylation changes in differentiating hESCs both hyper- and hypomethylated
